# Poly[[tetra-μ-cyanido-κ^8^ *C*:*N*-dodeca-cyanido-κ^12^ *C*-tris­(*N*,*N*-di­methyl­formamide-κ*O*)tris­(methanol-κ*O*)tris­(3,4,7,8-tetra­methyl-1,10-phenanthroline-κ^2^ *N*,*N*′)trimanganese(II)ditungstate(V)] dihydrate]

**DOI:** 10.1107/S1600536814007235

**Published:** 2014-04-05

**Authors:** Fei-Lin Yang, Dan Yang

**Affiliations:** a School of Environmental and Chemical Engineering, Jiangsu University of Science and Technology, Zhenjiang 212003, People’s Republic of China

## Abstract

The asymmetric unit of the title compound, {[Mn_3_{W(CN)_8_}_2_(C_16_H_16_N_2_)_3_(C_3_H_7_NO)_3_(CH_3_OH)_3_]·2H_2_O}_*n*_, consists of three [Mn(*N*,*N*-di­methyl­formamide)(methanol)(3,4,7,8-tetra­methyl-1,10-phenanthroline)]^2+^ cations, two [W(CN)_8_]^3−^ anions and two water mol­ecules. Each water mol­ecule is disordered over three sets of sites, with a refined occupancy ratio of 0.310 (9):0.275 (9):0.415 (9) for one mol­ecule and 0.335 (9):0.288 (9):0.377 (9) for the other mol­ecule. The Mn^II^ atoms exhibit a distorted octa­hedral geometry, while the W^V^ atoms adopt a distorted square-anti­prismatic geometry. The Mn^II^ and W^V^ atoms are linked alternatively through cyanide groups, forming a tetra­nuclear 12-atom rhombic metallacycle. Adjacent metallacycles are further connected by μ_2_-bridging cyanide anions, generating a 3,2-chain structure running parallel to [101]. Inter­chain π–π inter­actions are observed [centroid–centroid distances = 3.763 (3) and 3.620 (2) Å].

## Related literature   

For general background to octacyanidometalate-based compounds, see: Nowicka *et al.* (2012 ▶); Sieklucka *et al.* (2011 ▶). For related structures, see: Li *et al.* (2002 ▶, 2003 ▶); Withers *et al.* (2007 ▶). For the synthesis of octacyanidotungstate(V), see: Bok *et al.* (1975 ▶). 
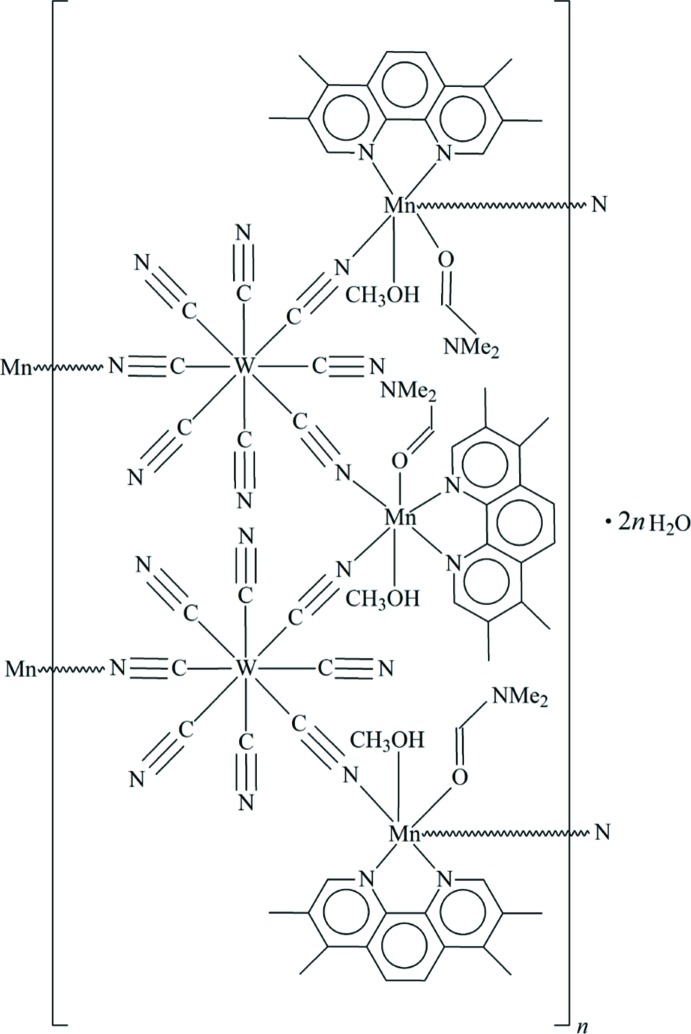


## Experimental   

### 

#### Crystal data  

[Mn_3_W_2_(CN)_16_(C_16_H_16_N_2_)_3_(C_3_H_7_NO)_3_(CH_4_O)_3_]·2H_2_O
*M* *_r_* = 2009.20
Triclinic, 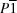

*a* = 15.5038 (17) Å
*b* = 16.4958 (18) Å
*c* = 21.4067 (13) Å
α = 70.008 (2)°
β = 82.663 (3)°
γ = 66.129 (2)°
*V* = 4704.4 (8) Å^3^
*Z* = 2
Mo *K*α radiation
μ = 2.89 mm^−1^
*T* = 291 K
0.24 × 0.20 × 0.18 mm

#### Data collection  

Bruker SMART APEXII diffractometer
Absorption correction: multi-scan (*SADABS*; Bruker, 2004 ▶) *T*_min_ = 0.50, *T*_max_ = 0.60
35664 measured reflections
17934 independent reflections
14773 reflections with *I* > 2σ(*I*)
*R*_int_ = 0.016

#### Refinement  

*R*[*F* ^2^ > 2σ(*F* ^2^)] = 0.046
*wR*(*F* ^2^) = 0.117
*S* = 1.05
17934 reflections
1078 parameters
2 restraints
H-atom parameters constrained
Δρ_max_ = 1.38 e Å^−3^
Δρ_min_ = −0.92 e Å^−3^

### 

Data collection: *APEX2* (Bruker, 2004 ▶); cell refinement: *SAINT* (Bruker, 2004 ▶); data reduction: *SAINT*; program(s) used to solve structure: *SHELXS97* (Sheldrick, 2008 ▶); program(s) used to refine structure: *SHELXL2013* (Sheldrick, 2008 ▶); molecular graphics: *DIAMOND* (Brandenburg, 2006 ▶); software used to prepare material for publication: *SHELXTL* (Sheldrick, 2008 ▶).

## Supplementary Material

Crystal structure: contains datablock(s) I, New_Global_Publ_Block. DOI: 10.1107/S1600536814007235/rz5113sup1.cif

Structure factors: contains datablock(s) I. DOI: 10.1107/S1600536814007235/rz5113Isup2.hkl

CCDC reference: 994877

Additional supporting information:  crystallographic information; 3D view; checkCIF report

## Figures and Tables

**Fig. 1.**
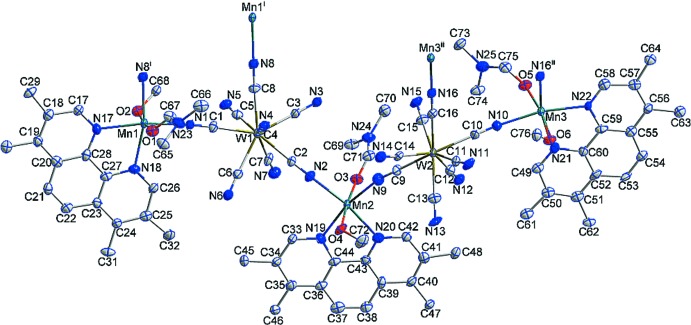
The molecular structure of the title compound with displacement ellipsoids drawn at the 30% probability level. H atoms and disordered water molecules are omitted for clarity. Symmetry code: (i) -x, -y + 2, -z; (ii) -x + 1, -y + 2, -z + 1.

**Fig. 2.**
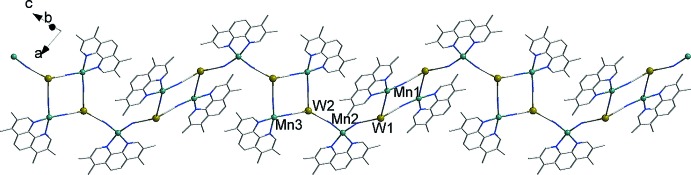
The 3,2-chain structure of the title compound. H atoms, coordinated methanol and DMF ligands, terminal cyanide groups, and water molecules are omitted for clarity.

## supplementary crystallographic information

### 1. Comment

Recently, increasing attention has been put into the design and syntheses of functional materials, among which octacyanometalates [*M*(CN)_8_]*^n^*^-^ (*M* = Mo, W; *n* = 3, 4) with flexible coordination modes and low symmetries have been widely studied (Nowicka *et al.*, 2012; Sieklucka *et al.*, 2011). For such systems, chelated ligands are usually employed to control on metal centers for CN-bridging, hence generating low-dimensional architectures rather than extended networks. As a part of a detailed study of cyano-bridged assemblies, we used [W(CN)_8_]^3-^ as precursor to react with Mn^2+^ ions and the chelated ligand 3,4,7,8-tetramethyl-1,10-phenanthroline (tmphen), and a new bimetallic compound with 3,2 chain structure was obtained.

The asymmetric unit of the title compound (Fig. 1) consists of three [Mn(3,4,7,8-tetramethyl-1,10-phenanthroline)(methanol)(*N*,*N*-dimethylformamide)]^2+^ cations, two [W(CN)_8_]^3-^ anions, and two disordered water molecules of crystallization. The Mn atoms exhibit a distorted octahedral geometry, where the coordination sites are occupied by two nitrogen atoms of one tmphen ligand, two bridging cyanide groups and two oxygen atoms of a DMF and a CH_3_OH molecule. The eight-coordinated W centres adopt a distorted square antiprismatic geometry, where three cyanide groups bridge to adjacent three Mn atoms and the others are terminal. As a result, the W1 and Mn1 atoms are connected alternatively by cyanide groups to form a tetranuclear 12-atom rhombic metallacycle, Mn1_2_W1_2_(CN)_4_, with the W1 and Mn1 centres located at the vertexes and the cyanide groups forming the sides. Similarly, another rhombic cycle, Mn3_2_W2_2_(CN)_4_, is also formed by the connection of W2 and Mn3 atoms through cyanide anions. The Mn2 atoms further link alternatively adjacent circles through –CN–Mn2—NC– linkages, generating a 3,2-chain structure parallel to [1 0 1]. Adjacent chains are linked by π-π interactions involving the phenanthroline rings systems, with centroid-to-centroid distances of 3.763 (3) and 3.620 (2) Å. The structural feature observed in the title compound has been also found in other octacyanometalate-based materials (Li *et al.*, 2002; Li *et al.*, 2003; Withers *et al.*, 2007).

### 2. Experimental

The title compound was prepared at room temperature by slow diffusion of a CH_3_OH/DMF solution (1:1 *v*/*v*) containing [HN(*n*-C_4_H_9_)_3_[W(CN)_8_] (0.05 mmol) (Bok *et al.*, 1975) and MnCl_2_·4H_2_O (0.15 mmol) into a methanol solution of tmphen (0.1 mmol). After about two weeks, brown block crystals were obtained on slow evaporation of the solvent.

### 3. Refinement

The two independent water molecule are disordered over three sets of sites with refined occupancy ratios of 0.310 (9):0.275 (9):0.415 (9) for one molecule and 0.335 (9):0.288 (9):0.377 (9) for the other molecule. The hydroxy and water H atoms were located in a difference Fourier map and refined as riding [O—H = 0.85 Å, *U*(H) = 1.5*U*_eq_(O) for water H atoms; O—H = 0.97 Å, *U*(H) = 1.2*U*_eq_(O) for hydroxy H atoms]. All other H atoms were calculated at idealized positions and included in the refinement in a riding mode, with C–H = 0.93-0.97 Å and with *U*(H) = 1.2*U*_eq_(C) or 1.5*U*_eq_(C) for methyl H atoms.

### Crystal data

**Table:**

[Mn_3_W_2_(CN)_16_(C_16_H_16_N_2_)_3_(C_3_H_7_NO)_3_(CH_4_O)_3_]·2H_2_O	*Z* = 2
*M**_r_* = 2009.20	*F*(000) = 2006
Triclinic, *P*1	*D*_x_ = 1.418 Mg m^−^^3^
Hall symbol: -P 1	Mo *K*α radiation, λ = 0.71073 Å
*a* = 15.5038 (17) Å	Cell parameters from 9991 reflections
*b* = 16.4958 (18) Å	θ = 2.5–27.3°
*c* = 21.4067 (13) Å	µ = 2.89 mm^−^^1^
α = 70.008 (2)°	*T* = 291 K
β = 82.663 (3)°	Block, brown
γ = 66.129 (2)°	0.24 × 0.20 × 0.18 mm
*V* = 4704.4 (8) Å^3^	

### Data collection

**Table:**

Bruker SMART APEXII diffractometer	17934 independent reflections
Radiation source: sealed tube	14773 reflections with *I* > 2σ(*I*)
Graphite monochromator	*R*_int_ = 0.016
phi and ω scans	θ_max_ = 26.0°, θ_min_ = 1.4°
Absorption correction: multi-scan (*SADABS*; Bruker, 2004)	*h* = −18→18
*T*_min_ = 0.50, *T*_max_ = 0.60	*k* = −20→20
35664 measured reflections	*l* = −26→26

### Refinement

**Table:**

Refinement on *F*^2^	2 restraints
Least-squares matrix: full	Hydrogen site location: mixed
*R*[*F*^2^ > 2σ(*F*^2^)] = 0.046	H-atom parameters constrained
*wR*(*F*^2^) = 0.117	*w* = 1/[σ^2^(*F*_o_^2^) + (0.07*P*)^2^ + 1.88*P*] where *P* = (*F*_o_^2^ + 2*F*_c_^2^)/3
*S* = 1.05	(Δ/σ)_max_ = 0.002
17934 reflections	Δρ_max_ = 1.38 e Å^−^^3^
1078 parameters	Δρ_min_ = −0.92 e Å^−^^3^

### Special details

**Table:**

Geometry. All e.s.d.'s (except the e.s.d. in the dihedral angle between two l.s. planes) are estimated using the full covariance matrix. The cell e.s.d.'s are taken into account individually in the estimation of e.s.d.'s in distances, angles and torsion angles; correlations between e.s.d.'s in cell parameters are only used when they are defined by crystal symmetry. An approximate (isotropic) treatment of cell e.s.d.'s is used for estimating e.s.d.'s involving l.s. planes.

### Fractional atomic coordinates and isotropic or equivalent isotropic displacement parameters (Å^2^)

**Table:**

	*x*	*y*	*z*	*U*_iso_*/*U*_eq_	Occ. (<1)
C1	0.1521 (3)	0.8254 (4)	0.0296 (3)	0.0359 (12)	
C2	0.3782 (3)	0.7348 (3)	0.1466 (2)	0.0310 (11)	
C3	0.3539 (4)	0.9040 (4)	0.0708 (3)	0.0429 (13)	
C4	0.1993 (4)	0.8160 (3)	0.1541 (3)	0.0354 (12)	
C5	0.2751 (4)	0.8947 (4)	−0.0439 (3)	0.0396 (13)	
C6	0.2926 (4)	0.6808 (4)	0.0810 (3)	0.0389 (12)	
C7	0.4185 (4)	0.7582 (4)	0.0219 (3)	0.0457 (14)	
C8	0.1791 (4)	0.9644 (4)	0.0506 (3)	0.0357 (12)	
C9	0.5504 (3)	0.7187 (4)	0.3507 (2)	0.0358 (12)	
C10	0.6241 (4)	0.8491 (4)	0.4541 (3)	0.0377 (12)	
C11	0.5441 (3)	0.7372 (3)	0.5347 (2)	0.0301 (11)	
C12	0.6920 (4)	0.7414 (3)	0.3850 (3)	0.0344 (11)	
C13	0.6214 (3)	0.6260 (4)	0.4708 (3)	0.0342 (11)	
C14	0.4292 (4)	0.7417 (3)	0.4451 (2)	0.0325 (11)	
C15	0.5299 (3)	0.8921 (4)	0.3384 (3)	0.0351 (11)	
C16	0.4397 (4)	0.8921 (4)	0.4495 (3)	0.0351 (11)	
C17	−0.2202 (4)	0.8318 (4)	−0.0874 (3)	0.0410 (13)	
H17	−0.2451	0.8893	−0.0800	0.049*	
C18	−0.2813 (4)	0.8061 (4)	−0.1164 (3)	0.0391 (12)	
C19	−0.2414 (4)	0.7213 (4)	−0.1306 (2)	0.0404 (13)	
C20	−0.1446 (4)	0.6640 (4)	−0.1161 (2)	0.0373 (12)	
C21	−0.0995 (4)	0.5785 (4)	−0.1275 (3)	0.0459 (14)	
H21	−0.1331	0.5579	−0.1472	0.055*	
C22	−0.0097 (4)	0.5256 (4)	−0.1110 (3)	0.0471 (14)	
H22	0.0183	0.4710	−0.1223	0.057*	
C23	0.0461 (4)	0.5482 (3)	−0.0768 (2)	0.0350 (11)	
C24	0.1423 (4)	0.4928 (3)	−0.0594 (3)	0.0403 (12)	
C25	0.1892 (4)	0.5245 (4)	−0.0273 (3)	0.0394 (12)	
C26	0.1365 (4)	0.6138 (4)	−0.0141 (3)	0.0395 (12)	
H26	0.1668	0.6342	0.0084	0.047*	
C27	0.0028 (3)	0.6366 (4)	−0.0651 (3)	0.0355 (11)	
C28	−0.0929 (4)	0.6942 (4)	−0.0845 (2)	0.0373 (12)	
C29	−0.3840 (4)	0.8691 (4)	−0.1280 (3)	0.0490 (15)	
H29A	−0.3984	0.8918	−0.1746	0.073*	
H29B	−0.3972	0.9210	−0.1125	0.073*	
H29C	−0.4218	0.8346	−0.1042	0.073*	
C30	−0.2991 (4)	0.6941 (4)	−0.1652 (3)	0.0499 (15)	
H30A	−0.3647	0.7325	−0.1636	0.075*	
H30B	−0.2892	0.6295	−0.1434	0.075*	
H30C	−0.2800	0.7029	−0.2107	0.075*	
C31	0.1926 (4)	0.4025 (4)	−0.0733 (3)	0.0524 (15)	
H31A	0.2113	0.4146	−0.1190	0.079*	
H31B	0.1514	0.3695	−0.0648	0.079*	
H31C	0.2475	0.3651	−0.0451	0.079*	
C32	0.2883 (4)	0.4722 (4)	−0.0034 (3)	0.0499 (15)	
H32A	0.2964	0.4102	0.0248	0.075*	
H32B	0.3032	0.5039	0.0213	0.075*	
H32C	0.3295	0.4688	−0.0407	0.075*	
C33	0.5531 (4)	0.4911 (4)	0.1545 (3)	0.0396 (13)	
H33	0.4911	0.5346	0.1478	0.048*	
C34	0.5804 (4)	0.4181 (4)	0.1271 (3)	0.0458 (14)	
C35	0.6719 (4)	0.3496 (4)	0.1393 (3)	0.0416 (13)	
C36	0.7347 (4)	0.3573 (4)	0.1769 (2)	0.0355 (11)	
C37	0.8265 (4)	0.2962 (4)	0.1901 (3)	0.0396 (12)	
H37	0.8478	0.2457	0.1741	0.048*	
C38	0.8898 (4)	0.3034 (4)	0.2252 (3)	0.0468 (14)	
H38	0.9518	0.2602	0.2315	0.056*	
C39	0.8564 (3)	0.3825 (4)	0.2529 (2)	0.0345 (11)	
C40	0.9163 (4)	0.3951 (4)	0.2905 (3)	0.0391 (12)	
C41	0.8787 (4)	0.4701 (4)	0.3136 (2)	0.0357 (11)	
C42	0.7823 (4)	0.5281 (4)	0.3031 (3)	0.0384 (12)	
H42	0.7566	0.5759	0.3219	0.046*	
C43	0.7631 (3)	0.4459 (3)	0.2411 (2)	0.0314 (11)	
C44	0.7007 (3)	0.4341 (3)	0.2017 (2)	0.0321 (11)	
C45	0.5094 (4)	0.4124 (4)	0.0877 (3)	0.0490 (15)	
H45A	0.5329	0.4133	0.0439	0.073*	
H45B	0.4507	0.4648	0.0847	0.073*	
H45C	0.4998	0.3554	0.1098	0.073*	
C46	0.7036 (4)	0.2726 (4)	0.1097 (3)	0.0451 (14)	
H46A	0.6978	0.2180	0.1419	0.068*	
H46B	0.7683	0.2585	0.0967	0.068*	
H46C	0.6652	0.2919	0.0714	0.068*	
C47	1.0180 (4)	0.3308 (4)	0.3010 (3)	0.0486 (15)	
H47A	1.0508	0.3560	0.3195	0.073*	
H47B	1.0454	0.3246	0.2592	0.073*	
H47C	1.0232	0.2705	0.3310	0.073*	
C48	0.9371 (4)	0.4916 (4)	0.3514 (3)	0.0416 (13)	
H48A	0.9800	0.4343	0.3807	0.062*	
H48B	0.8965	0.5299	0.3770	0.062*	
H48C	0.9721	0.5244	0.3208	0.062*	
C49	0.9022 (4)	0.7775 (3)	0.4635 (2)	0.0354 (11)	
H49	0.8532	0.7790	0.4417	0.042*	
C50	0.9923 (3)	0.7061 (4)	0.4656 (2)	0.0333 (11)	
C51	1.0648 (4)	0.7027 (4)	0.4979 (3)	0.0420 (13)	
C52	1.0468 (2)	0.7745 (2)	0.52851 (17)	0.0415 (13)	
C53	1.11751 (17)	0.7759 (2)	0.56158 (18)	0.0437 (14)	
H53	1.1782	0.7304	0.5644	0.052*	
C54	1.09746 (18)	0.8453 (2)	0.59036 (16)	0.0388 (12)	
H54	1.1448	0.8462	0.6125	0.047*	
C55	1.0067 (2)	0.9133 (2)	0.58607 (16)	0.0323 (11)	
C59	0.93601 (15)	0.9120 (2)	0.55300 (17)	0.0328 (11)	
C60	0.95606 (18)	0.8426 (2)	0.52422 (17)	0.0356 (12)	
C56	0.9821 (4)	0.9883 (4)	0.6154 (3)	0.0386 (12)	
C57	0.8944 (3)	1.0571 (4)	0.6045 (2)	0.0335 (11)	
C58	0.8272 (4)	1.0498 (4)	0.5679 (3)	0.0371 (12)	
H58	0.7672	1.0970	0.5604	0.045*	
C61	1.0044 (4)	0.6359 (4)	0.4306 (3)	0.0426 (13)	
H61A	1.0507	0.6388	0.3964	0.064*	
H61B	0.9453	0.6507	0.4111	0.064*	
H61C	1.0249	0.5740	0.4623	0.064*	
C62	1.1618 (4)	0.6307 (4)	0.4991 (3)	0.0408 (13)	
H62A	1.1618	0.5900	0.4760	0.061*	
H62B	1.1819	0.5948	0.5443	0.061*	
H62C	1.2041	0.6609	0.4777	0.061*	
C63	1.0555 (4)	0.9869 (4)	0.6562 (3)	0.0439 (13)	
H63A	1.0461	1.0499	0.6515	0.066*	
H63B	1.1173	0.9560	0.6410	0.066*	
H63C	1.0498	0.9538	0.7021	0.066*	
C64	0.8631 (4)	1.1363 (4)	0.6303 (3)	0.0445 (13)	
H64A	0.8487	1.1161	0.6766	0.067*	
H64B	0.8079	1.1854	0.6062	0.067*	
H64C	0.9125	1.1591	0.6250	0.067*	
C65	−0.1503 (5)	0.7056 (5)	0.2047 (4)	0.070 (2)	
H65A	−0.1856	0.7061	0.1707	0.106*	
H65B	−0.1891	0.7108	0.2429	0.106*	
H65C	−0.0957	0.6481	0.2168	0.106*	
C66	−0.1321 (6)	0.8402 (5)	0.2256 (3)	0.069 (2)	
H66A	−0.0717	0.8377	0.2342	0.103*	
H66B	−0.1588	0.8142	0.2667	0.103*	
H66C	−0.1733	0.9041	0.2052	0.103*	
C67	−0.0962 (5)	0.8135 (4)	0.1114 (3)	0.0544 (16)	
H67	−0.0901	0.8709	0.0949	0.065*	
C68	0.0515 (3)	0.9458 (4)	−0.1466 (3)	0.0381 (12)	
H68A	−0.0014	1.0033	−0.1492	0.057*	
H68B	0.0816	0.9508	−0.1890	0.057*	
H68C	0.0956	0.9339	−0.1137	0.057*	
C69	0.6410 (5)	0.8428 (4)	0.0452 (3)	0.0575 (17)	
H69A	0.7080	0.8242	0.0395	0.086*	
H69B	0.6091	0.8997	0.0103	0.086*	
H69C	0.6228	0.7945	0.0435	0.086*	
C70	0.6049 (4)	0.9500 (4)	0.1132 (3)	0.0505 (15)	
H70A	0.5856	0.9536	0.1569	0.076*	
H70B	0.5581	0.9990	0.0812	0.076*	
H70C	0.6642	0.9569	0.1033	0.076*	
C71	0.6026 (4)	0.7898 (4)	0.1670 (3)	0.0419 (13)	
H71	0.5832	0.8049	0.2060	0.050*	
C72	0.5586 (6)	0.4704 (6)	0.3905 (3)	0.076 (2)	
H72A	0.6139	0.4141	0.3948	0.114*	
H72B	0.5104	0.4558	0.4193	0.114*	
H72C	0.5739	0.5139	0.4027	0.114*	
C73	0.5550 (4)	1.1644 (4)	0.2629 (3)	0.0566 (16)	
H73A	0.5153	1.2081	0.2849	0.085*	
H73B	0.5216	1.1293	0.2573	0.085*	
H73C	0.5720	1.1977	0.2201	0.085*	
C74	0.7089 (4)	1.0296 (5)	0.2743 (3)	0.0578 (17)	
H74A	0.7720	1.0144	0.2872	0.087*	
H74B	0.7029	1.0549	0.2267	0.087*	
H74C	0.6954	0.9741	0.2895	0.087*	
C75	0.6556 (4)	1.0941 (4)	0.3687 (3)	0.0411 (13)	
H75	0.6065	1.1348	0.3866	0.049*	
C76	0.6534 (4)	0.8406 (4)	0.6353 (3)	0.0442 (13)	
H76A	0.6257	0.8946	0.6498	0.066*	
H76B	0.6650	0.7850	0.6731	0.066*	
H76C	0.6109	0.8435	0.6049	0.066*	
Mn1	−0.03069 (5)	0.81206 (5)	−0.02729 (3)	0.02748 (16)	
Mn2	0.56980 (5)	0.60947 (5)	0.24169 (3)	0.02728 (16)	
Mn3	0.74155 (5)	0.94644 (5)	0.50428 (4)	0.02783 (16)	
N1	0.0866 (3)	0.8243 (3)	0.0120 (2)	0.0336 (9)	
N2	0.4310 (3)	0.6892 (3)	0.1902 (2)	0.0380 (10)	
N3	0.3941 (3)	0.9495 (3)	0.0763 (2)	0.0441 (11)	
N4	0.1516 (3)	0.8172 (3)	0.1998 (2)	0.0467 (12)	
N5	0.2666 (3)	0.9319 (3)	−0.0990 (2)	0.0387 (10)	
N6	0.2984 (3)	0.6095 (3)	0.0904 (2)	0.0465 (12)	
N7	0.4890 (3)	0.7237 (3)	0.0028 (2)	0.0462 (11)	
N8	0.1279 (3)	1.0414 (3)	0.04165 (19)	0.0287 (9)	
N9	0.5485 (3)	0.6895 (3)	0.3092 (2)	0.0389 (10)	
N10	0.6631 (3)	0.8854 (3)	0.4688 (2)	0.0378 (10)	
N11	0.5381 (3)	0.7220 (3)	0.5902 (2)	0.0447 (11)	
N12	0.7669 (3)	0.7213 (3)	0.3624 (2)	0.0446 (11)	
N13	0.6539 (3)	0.5467 (3)	0.4967 (2)	0.0537 (14)	
N14	0.3619 (3)	0.7270 (3)	0.4550 (2)	0.0439 (11)	
N15	0.5146 (3)	0.9545 (3)	0.2906 (2)	0.0469 (12)	
N16	0.3781 (3)	0.9527 (3)	0.4608 (2)	0.0380 (10)	
N17	−0.1326 (3)	0.7784 (3)	−0.0715 (2)	0.0351 (10)	
N18	0.0488 (3)	0.6653 (3)	−0.0330 (2)	0.0369 (10)	
N19	0.6115 (3)	0.5017 (3)	0.1901 (2)	0.0362 (10)	
N20	0.7263 (3)	0.5166 (3)	0.26685 (19)	0.0342 (10)	
N21	0.8853 (3)	0.8417 (3)	0.4914 (2)	0.0353 (10)	
N22	0.8461 (3)	0.9800 (3)	0.5443 (2)	0.0334 (9)	
N23	−0.1205 (4)	0.7845 (4)	0.1795 (3)	0.0560 (13)	
N24	0.6151 (3)	0.8585 (3)	0.1104 (2)	0.0462 (11)	
N25	0.6421 (3)	1.0991 (3)	0.3038 (2)	0.0452 (11)	
O1	−0.0828 (3)	0.7703 (3)	0.07290 (18)	0.0450 (9)	
O2	0.0193 (2)	0.8697 (2)	−0.12824 (16)	0.0358 (8)	
H2B	−0.0315	0.8889	−0.1590	0.043*	
O3	0.6160 (3)	0.7103 (2)	0.16782 (18)	0.0409 (9)	
O4	0.5258 (3)	0.5103 (3)	0.32424 (17)	0.0441 (9)	
H4B	0.5367	0.4582	0.3087	0.053*	
O5	0.7262 (2)	1.0410 (3)	0.40470 (17)	0.0413 (9)	
O6	0.7408 (3)	0.8384 (3)	0.60266 (18)	0.0430 (9)	
H6B	0.7790	0.8422	0.6333	0.052*	
O1W	0.1213 (11)	0.5827 (10)	0.2403 (7)	0.068 (5)	0.310 (9)
H1WA	0.0723	0.5730	0.2375	0.101*	0.310 (9)
H1WC	0.1161	0.6025	0.2729	0.101*	0.310 (9)
O2W	0.6638 (11)	0.2913 (11)	0.3608 (8)	0.060 (5)	0.275 (9)
H2WA	0.6680	0.2473	0.3970	0.090*	0.275 (9)
H2WB	0.6954	0.2685	0.3310	0.090*	0.275 (9)
O3W	0.6552 (8)	0.6032 (7)	0.7210 (5)	0.061 (4)	0.415 (9)
H3WA	0.7098	0.5793	0.7385	0.092*	0.415 (9)
H3WC	0.6157	0.5928	0.7508	0.092*	0.415 (9)
O4W	0.8202 (8)	0.6538 (7)	0.6107 (5)	0.042 (3)	0.335 (9)
H4WD	0.7795	0.6403	0.6381	0.050*	0.335 (9)
H4WB	0.8648	0.6038	0.6082	0.062*	0.335 (9)
O5W	0.2852 (10)	0.6327 (9)	0.3382 (6)	0.049 (4)	0.288 (9)
H5WA	0.2558	0.6154	0.3178	0.073*	0.288 (9)
H5WB	0.2457	0.6725	0.3554	0.073*	0.288 (9)
O6W	0.7496 (6)	1.0023 (5)	0.7488 (4)	0.028 (3)	0.377 (9)
H6WB	0.6909	1.0140	0.7492	0.042*	0.377 (9)
H6WC	0.7735	0.9904	0.7134	0.042*	0.377 (9)
W1	0.28113 (2)	0.82252 (2)	0.06363 (2)	0.03084 (7)	
W2	0.55316 (2)	0.77498 (2)	0.42800 (2)	0.02667 (7)	

### Atomic displacement parameters (Å^2^)

**Table:**

	*U*^11^	*U*^22^	*U*^33^	*U*^12^	*U*^13^	*U*^23^
C1	0.025 (3)	0.046 (3)	0.041 (3)	−0.011 (2)	−0.006 (2)	−0.020 (2)
C2	0.026 (2)	0.039 (3)	0.031 (3)	−0.015 (2)	−0.006 (2)	−0.010 (2)
C3	0.037 (3)	0.037 (3)	0.048 (3)	−0.015 (2)	−0.020 (3)	0.003 (3)
C4	0.033 (3)	0.034 (3)	0.029 (3)	−0.006 (2)	−0.013 (2)	−0.003 (2)
C5	0.049 (3)	0.035 (3)	0.032 (3)	−0.011 (3)	−0.008 (2)	−0.009 (2)
C6	0.032 (3)	0.040 (3)	0.046 (3)	−0.012 (2)	−0.009 (2)	−0.015 (3)
C7	0.028 (3)	0.068 (4)	0.037 (3)	−0.012 (3)	−0.012 (2)	−0.016 (3)
C8	0.036 (3)	0.034 (3)	0.041 (3)	−0.020 (2)	0.004 (2)	−0.008 (2)
C9	0.033 (3)	0.044 (3)	0.033 (3)	−0.010 (2)	−0.002 (2)	−0.020 (2)
C10	0.042 (3)	0.036 (3)	0.038 (3)	−0.021 (2)	−0.008 (2)	−0.005 (2)
C11	0.031 (3)	0.033 (3)	0.030 (3)	−0.014 (2)	−0.005 (2)	−0.012 (2)
C12	0.031 (3)	0.037 (3)	0.034 (3)	−0.011 (2)	−0.007 (2)	−0.011 (2)
C13	0.028 (3)	0.035 (3)	0.035 (3)	−0.006 (2)	0.006 (2)	−0.015 (2)
C14	0.032 (3)	0.025 (2)	0.033 (3)	−0.003 (2)	−0.013 (2)	−0.006 (2)
C15	0.030 (3)	0.035 (3)	0.034 (3)	−0.008 (2)	0.002 (2)	−0.009 (2)
C16	0.031 (3)	0.034 (3)	0.039 (3)	−0.007 (2)	−0.008 (2)	−0.014 (2)
C17	0.027 (3)	0.044 (3)	0.054 (4)	−0.014 (2)	−0.003 (2)	−0.017 (3)
C18	0.034 (3)	0.055 (3)	0.032 (3)	−0.027 (3)	−0.002 (2)	−0.006 (2)
C19	0.052 (3)	0.048 (3)	0.028 (3)	−0.030 (3)	−0.007 (2)	−0.004 (2)
C20	0.048 (3)	0.049 (3)	0.023 (3)	−0.026 (3)	0.001 (2)	−0.013 (2)
C21	0.059 (4)	0.057 (4)	0.039 (3)	−0.036 (3)	0.002 (3)	−0.021 (3)
C22	0.053 (4)	0.051 (3)	0.039 (3)	−0.021 (3)	−0.005 (3)	−0.013 (3)
C23	0.043 (3)	0.033 (3)	0.032 (3)	−0.017 (2)	0.004 (2)	−0.012 (2)
C24	0.048 (3)	0.027 (3)	0.040 (3)	−0.009 (2)	−0.005 (2)	−0.009 (2)
C25	0.043 (3)	0.038 (3)	0.032 (3)	−0.013 (2)	−0.005 (2)	−0.006 (2)
C26	0.039 (3)	0.036 (3)	0.040 (3)	−0.016 (2)	−0.009 (2)	−0.002 (2)
C27	0.030 (3)	0.038 (3)	0.034 (3)	−0.011 (2)	0.006 (2)	−0.011 (2)
C28	0.038 (3)	0.051 (3)	0.033 (3)	−0.023 (3)	0.003 (2)	−0.020 (2)
C29	0.032 (3)	0.071 (4)	0.034 (3)	−0.017 (3)	−0.001 (2)	−0.008 (3)
C30	0.045 (3)	0.065 (4)	0.047 (4)	−0.027 (3)	−0.002 (3)	−0.019 (3)
C31	0.064 (4)	0.037 (3)	0.052 (4)	−0.019 (3)	0.019 (3)	−0.017 (3)
C32	0.049 (3)	0.040 (3)	0.055 (4)	−0.022 (3)	−0.007 (3)	0.001 (3)
C33	0.033 (3)	0.041 (3)	0.047 (3)	−0.009 (2)	−0.017 (2)	−0.016 (3)
C34	0.049 (3)	0.037 (3)	0.045 (3)	−0.005 (3)	−0.019 (3)	−0.013 (3)
C35	0.057 (4)	0.030 (3)	0.033 (3)	−0.014 (3)	−0.017 (3)	−0.001 (2)
C36	0.038 (3)	0.037 (3)	0.032 (3)	−0.012 (2)	0.004 (2)	−0.015 (2)
C37	0.036 (3)	0.044 (3)	0.032 (3)	−0.014 (3)	0.012 (2)	−0.010 (2)
C38	0.033 (3)	0.049 (3)	0.058 (4)	−0.014 (3)	0.007 (3)	−0.021 (3)
C39	0.032 (3)	0.044 (3)	0.029 (3)	−0.021 (2)	−0.002 (2)	−0.007 (2)
C40	0.033 (3)	0.046 (3)	0.034 (3)	−0.011 (2)	−0.006 (2)	−0.010 (2)
C41	0.036 (3)	0.041 (3)	0.029 (3)	−0.020 (2)	0.002 (2)	−0.004 (2)
C42	0.035 (3)	0.049 (3)	0.035 (3)	−0.019 (2)	−0.003 (2)	−0.013 (2)
C43	0.030 (3)	0.032 (3)	0.035 (3)	−0.016 (2)	0.005 (2)	−0.011 (2)
C44	0.031 (3)	0.030 (3)	0.031 (3)	−0.010 (2)	0.000 (2)	−0.007 (2)
C45	0.048 (3)	0.038 (3)	0.067 (4)	−0.012 (3)	−0.019 (3)	−0.023 (3)
C46	0.049 (3)	0.034 (3)	0.056 (4)	−0.012 (3)	−0.012 (3)	−0.019 (3)
C47	0.041 (3)	0.037 (3)	0.050 (4)	−0.003 (3)	−0.016 (3)	−0.001 (3)
C48	0.030 (3)	0.052 (3)	0.039 (3)	−0.018 (3)	−0.007 (2)	−0.005 (3)
C49	0.032 (3)	0.036 (3)	0.030 (3)	−0.006 (2)	0.002 (2)	−0.009 (2)
C50	0.025 (2)	0.043 (3)	0.029 (3)	−0.015 (2)	0.019 (2)	−0.013 (2)
C51	0.026 (3)	0.052 (3)	0.037 (3)	−0.007 (2)	0.005 (2)	−0.012 (3)
C52	0.033 (3)	0.027 (3)	0.049 (3)	−0.003 (2)	−0.010 (2)	−0.001 (2)
C53	0.035 (3)	0.042 (3)	0.040 (3)	0.006 (2)	−0.011 (2)	−0.018 (3)
C54	0.023 (3)	0.047 (3)	0.037 (3)	−0.005 (2)	−0.007 (2)	−0.011 (2)
C55	0.023 (2)	0.044 (3)	0.022 (2)	−0.009 (2)	0.0001 (19)	−0.007 (2)
C59	0.018 (2)	0.039 (3)	0.037 (3)	−0.009 (2)	0.008 (2)	−0.013 (2)
C60	0.027 (3)	0.042 (3)	0.040 (3)	−0.016 (2)	−0.001 (2)	−0.012 (2)
C56	0.037 (3)	0.035 (3)	0.035 (3)	−0.009 (2)	−0.006 (2)	−0.005 (2)
C57	0.035 (3)	0.038 (3)	0.030 (3)	−0.018 (2)	0.011 (2)	−0.013 (2)
C58	0.036 (3)	0.043 (3)	0.039 (3)	−0.018 (2)	0.005 (2)	−0.020 (2)
C61	0.041 (3)	0.040 (3)	0.043 (3)	−0.010 (3)	0.008 (2)	−0.018 (3)
C62	0.032 (3)	0.043 (3)	0.035 (3)	−0.006 (2)	0.010 (2)	−0.011 (2)
C63	0.043 (3)	0.048 (3)	0.043 (3)	−0.024 (3)	0.000 (3)	−0.009 (3)
C64	0.035 (3)	0.054 (3)	0.047 (3)	−0.014 (3)	0.003 (2)	−0.025 (3)
C65	0.083 (5)	0.069 (5)	0.070 (5)	−0.047 (4)	0.004 (4)	−0.015 (4)
C66	0.092 (6)	0.077 (5)	0.050 (4)	−0.043 (4)	0.023 (4)	−0.030 (4)
C67	0.082 (5)	0.048 (3)	0.036 (3)	−0.029 (3)	0.010 (3)	−0.015 (3)
C68	0.025 (3)	0.045 (3)	0.042 (3)	−0.012 (2)	−0.007 (2)	−0.011 (3)
C69	0.058 (4)	0.054 (4)	0.047 (4)	−0.013 (3)	0.016 (3)	−0.016 (3)
C70	0.042 (3)	0.062 (4)	0.054 (4)	−0.029 (3)	0.011 (3)	−0.018 (3)
C71	0.042 (3)	0.053 (4)	0.030 (3)	−0.016 (3)	−0.001 (2)	−0.014 (3)
C72	0.097 (6)	0.085 (5)	0.038 (4)	−0.050 (5)	−0.012 (4)	0.012 (4)
C73	0.037 (3)	0.057 (4)	0.053 (4)	−0.007 (3)	−0.007 (3)	−0.002 (3)
C74	0.054 (4)	0.062 (4)	0.038 (3)	−0.001 (3)	0.003 (3)	−0.021 (3)
C75	0.031 (3)	0.050 (3)	0.042 (3)	−0.013 (3)	0.012 (2)	−0.022 (3)
C76	0.045 (3)	0.067 (4)	0.029 (3)	−0.029 (3)	0.002 (2)	−0.017 (3)
Mn1	0.0281 (4)	0.0317 (4)	0.0259 (4)	−0.0128 (3)	−0.0077 (3)	−0.0086 (3)
Mn2	0.0288 (4)	0.0307 (4)	0.0254 (4)	−0.0115 (3)	−0.0082 (3)	−0.0098 (3)
Mn3	0.0246 (4)	0.0327 (4)	0.0286 (4)	−0.0085 (3)	−0.0055 (3)	−0.0136 (3)
N1	0.025 (2)	0.040 (2)	0.035 (2)	−0.0101 (18)	−0.0076 (18)	−0.0114 (19)
N2	0.031 (2)	0.042 (3)	0.036 (2)	−0.010 (2)	−0.006 (2)	−0.009 (2)
N3	0.038 (3)	0.045 (3)	0.047 (3)	−0.019 (2)	−0.007 (2)	−0.006 (2)
N4	0.046 (3)	0.042 (3)	0.031 (3)	−0.001 (2)	−0.002 (2)	−0.005 (2)
N5	0.037 (2)	0.039 (3)	0.033 (3)	−0.015 (2)	0.002 (2)	−0.004 (2)
N6	0.042 (3)	0.038 (3)	0.056 (3)	−0.006 (2)	−0.017 (2)	−0.017 (2)
N7	0.035 (3)	0.057 (3)	0.048 (3)	−0.016 (2)	0.007 (2)	−0.024 (2)
N8	0.031 (2)	0.034 (2)	0.026 (2)	−0.0136 (19)	−0.0083 (17)	−0.0106 (18)
N9	0.038 (2)	0.059 (3)	0.024 (2)	−0.018 (2)	0.0104 (18)	−0.024 (2)
N10	0.030 (2)	0.036 (2)	0.054 (3)	−0.0117 (19)	−0.007 (2)	−0.021 (2)
N11	0.042 (3)	0.055 (3)	0.027 (3)	−0.025 (2)	0.000 (2)	0.008 (2)
N12	0.036 (3)	0.054 (3)	0.036 (3)	−0.013 (2)	−0.002 (2)	−0.009 (2)
N13	0.041 (3)	0.047 (3)	0.043 (3)	0.018 (2)	−0.008 (2)	−0.018 (2)
N14	0.036 (3)	0.046 (3)	0.046 (3)	−0.017 (2)	0.003 (2)	−0.010 (2)
N15	0.051 (3)	0.040 (3)	0.037 (3)	−0.014 (2)	−0.003 (2)	0.000 (2)
N16	0.030 (2)	0.037 (2)	0.045 (3)	−0.005 (2)	−0.0068 (19)	−0.017 (2)
N17	0.031 (2)	0.043 (2)	0.036 (2)	−0.017 (2)	−0.0013 (18)	−0.015 (2)
N18	0.038 (3)	0.037 (2)	0.035 (2)	−0.011 (2)	0.0029 (19)	−0.015 (2)
N19	0.035 (2)	0.041 (2)	0.038 (2)	−0.0100 (19)	−0.0023 (19)	−0.023 (2)
N20	0.031 (2)	0.046 (3)	0.023 (2)	−0.0094 (19)	0.0031 (17)	−0.0163 (19)
N21	0.018 (2)	0.043 (2)	0.043 (3)	−0.0078 (18)	0.0027 (18)	−0.019 (2)
N22	0.0155 (19)	0.036 (2)	0.044 (3)	−0.0046 (17)	−0.0061 (17)	−0.012 (2)
N23	0.073 (4)	0.056 (3)	0.046 (3)	−0.032 (3)	0.014 (3)	−0.020 (3)
N24	0.045 (3)	0.044 (3)	0.044 (3)	−0.013 (2)	0.006 (2)	−0.015 (2)
N25	0.036 (3)	0.049 (3)	0.043 (3)	−0.013 (2)	0.004 (2)	−0.013 (2)
O1	0.048 (2)	0.056 (2)	0.036 (2)	−0.025 (2)	0.0110 (17)	−0.0169 (19)
O2	0.0311 (19)	0.046 (2)	0.0271 (18)	−0.0147 (16)	0.0024 (14)	−0.0084 (16)
O3	0.045 (2)	0.036 (2)	0.040 (2)	−0.0166 (17)	0.0114 (17)	−0.0139 (17)
O4	0.043 (2)	0.048 (2)	0.0297 (19)	−0.0183 (18)	−0.0015 (16)	0.0025 (17)
O5	0.032 (2)	0.049 (2)	0.033 (2)	−0.0107 (17)	0.0025 (16)	−0.0085 (17)
O6	0.042 (2)	0.045 (2)	0.038 (2)	−0.0149 (18)	0.0020 (17)	−0.0115 (17)
O1W	0.075 (11)	0.060 (10)	0.062 (10)	−0.031 (8)	0.007 (8)	−0.010 (8)
O2W	0.066 (11)	0.059 (10)	0.056 (10)	−0.021 (8)	0.019 (8)	−0.030 (8)
O3W	0.072 (8)	0.070 (8)	0.044 (6)	−0.031 (6)	0.017 (5)	−0.023 (5)
O4W	0.040 (7)	0.032 (6)	0.043 (7)	−0.009 (5)	−0.017 (5)	0.001 (5)
O5W	0.052 (9)	0.042 (8)	0.038 (8)	−0.007 (7)	0.021 (6)	−0.017 (6)
O6W	0.027 (5)	0.030 (5)	0.027 (5)	−0.016 (4)	−0.012 (4)	0.001 (4)
W1	0.02969 (12)	0.03556 (12)	0.02926 (12)	−0.01160 (9)	−0.00575 (8)	−0.01186 (9)
W2	0.02480 (11)	0.03159 (11)	0.02509 (11)	−0.00796 (8)	−0.00767 (8)	−0.01161 (8)

### Geometric parameters (Å, º)

**Table:**

C1—N1	1.138 (6)	C51—C62	1.489 (7)
C1—W1	2.192 (5)	C52—C53	1.3900
C2—N2	1.152 (6)	C52—C60	1.3900
C2—W1	2.162 (5)	C53—C54	1.3900
C3—N3	1.193 (6)	C53—H53	0.9300
C3—W1	2.125 (5)	C54—C55	1.3900
C4—N4	1.149 (7)	C54—H54	0.9300
C4—W1	2.172 (6)	C55—C59	1.3900
C5—N5	1.125 (6)	C55—C56	1.468 (6)
C5—W1	2.193 (5)	C59—N22	1.377 (4)
C6—N6	1.091 (6)	C59—C60	1.3900
C6—W1	2.173 (6)	C60—N21	1.384 (4)
C7—N7	1.107 (7)	C56—C57	1.358 (7)
C7—W1	2.190 (6)	C56—C63	1.510 (7)
C8—N8	1.156 (6)	C57—C58	1.444 (7)
C8—W1	2.174 (5)	C57—C64	1.470 (7)
C9—N9	1.154 (6)	C58—N22	1.323 (6)
C9—W2	2.168 (5)	C58—H58	0.9300
C10—N10	1.137 (6)	C61—H61A	0.9600
C10—W2	2.175 (5)	C61—H61B	0.9600
C11—N11	1.127 (6)	C61—H61C	0.9600
C11—W2	2.154 (5)	C62—H62A	0.9600
C12—N12	1.160 (6)	C62—H62B	0.9600
C12—W2	2.161 (5)	C62—H62C	0.9600
C13—N13	1.147 (7)	C63—H63A	0.9600
C13—W2	2.145 (5)	C63—H63B	0.9600
C14—N14	1.143 (6)	C63—H63C	0.9600
C14—W2	2.160 (5)	C64—H64A	0.9600
C15—N15	1.145 (6)	C64—H64B	0.9600
C15—W2	2.149 (5)	C64—H64C	0.9600
C16—N16	1.142 (6)	C65—N23	1.463 (8)
C16—W2	2.166 (5)	C65—H65A	0.9600
C17—N17	1.296 (6)	C65—H65B	0.9600
C17—C18	1.455 (7)	C65—H65C	0.9600
C17—H17	0.9300	C66—N23	1.514 (8)
C18—C19	1.406 (8)	C66—H66A	0.9600
C18—C29	1.503 (7)	C66—H66B	0.9600
C19—C20	1.419 (8)	C66—H66C	0.9600
C19—C30	1.507 (7)	C67—O1	1.212 (6)
C20—C21	1.390 (8)	C67—N23	1.424 (7)
C20—C28	1.427 (7)	C67—H67	0.9300
C21—C22	1.324 (8)	C68—O2	1.449 (6)
C21—H21	0.9300	C68—H68A	0.9600
C22—C23	1.425 (7)	C68—H68B	0.9600
C22—H22	0.9300	C68—H68C	0.9600
C23—C24	1.417 (7)	C69—N24	1.482 (7)
C23—C27	1.434 (7)	C69—H69A	0.9600
C24—C25	1.397 (7)	C69—H69B	0.9600
C24—C31	1.490 (7)	C69—H69C	0.9600
C25—C26	1.472 (7)	C70—N24	1.473 (7)
C25—C32	1.482 (7)	C70—H70A	0.9600
C26—N18	1.305 (6)	C70—H70B	0.9600
C26—H26	0.9300	C70—H70C	0.9600
C27—N18	1.350 (6)	C71—O3	1.237 (6)
C27—C28	1.422 (7)	C71—N24	1.403 (7)
C28—N17	1.384 (6)	C71—H71	0.9300
C29—H29A	0.9600	C72—O4	1.407 (7)
C29—H29B	0.9600	C72—H72A	0.9600
C29—H29C	0.9600	C72—H72B	0.9600
C30—H30A	0.9600	C72—H72C	0.9600
C30—H30B	0.9600	C73—N25	1.494 (7)
C30—H30C	0.9600	C73—H73A	0.9600
C31—H31A	0.9600	C73—H73B	0.9600
C31—H31B	0.9600	C73—H73C	0.9600
C31—H31C	0.9600	C74—N25	1.467 (7)
C32—H32A	0.9600	C74—H74A	0.9600
C32—H32B	0.9600	C74—H74B	0.9600
C32—H32C	0.9600	C74—H74C	0.9600
C33—N19	1.351 (6)	C75—O5	1.241 (6)
C33—C34	1.409 (7)	C75—N25	1.401 (7)
C33—H33	0.9300	C75—H75	0.9300
C34—C35	1.397 (8)	C76—O6	1.437 (6)
C34—C45	1.518 (7)	C76—H76A	0.9600
C35—C36	1.406 (7)	C76—H76B	0.9600
C35—C46	1.491 (7)	C76—H76C	0.9600
C36—C37	1.367 (7)	Mn1—O1	2.174 (4)
C36—C44	1.419 (7)	Mn1—N1	2.203 (4)
C37—C38	1.372 (8)	Mn1—N8^i^	2.207 (4)
C37—H37	0.9300	Mn1—O2	2.222 (3)
C38—C39	1.492 (7)	Mn1—N18	2.269 (4)
C38—H38	0.9300	Mn1—N17	2.270 (4)
C39—C43	1.392 (7)	Mn2—O3	2.158 (4)
C39—C40	1.420 (7)	Mn2—N9	2.185 (4)
C40—C41	1.369 (7)	Mn2—O4	2.210 (3)
C40—C47	1.496 (7)	Mn2—N2	2.213 (4)
C41—C42	1.408 (7)	Mn2—N19	2.242 (4)
C41—C48	1.497 (7)	Mn2—N20	2.299 (4)
C42—N20	1.341 (6)	Mn3—O5	2.142 (4)
C42—H42	0.9300	Mn3—N16^ii^	2.183 (4)
C43—N20	1.345 (6)	Mn3—N10	2.196 (4)
C43—C44	1.470 (7)	Mn3—O6	2.248 (4)
C44—N19	1.368 (6)	Mn3—N22	2.253 (4)
C45—H45A	0.9600	Mn3—N21	2.254 (4)
C45—H45B	0.9600	N8—Mn1^i^	2.208 (4)
C45—H45C	0.9600	N16—Mn3^ii^	2.183 (4)
C46—H46A	0.9600	O2—H2B	0.9700
C46—H46B	0.9600	O4—H4B	0.9700
C46—H46C	0.9600	O6—H6B	0.9700
C47—H47A	0.9600	O1W—H1WA	0.8500
C47—H47B	0.9600	O1W—H1WC	0.8500
C47—H47C	0.9600	O2W—H2WA	0.8500
C48—H48A	0.9600	O2W—H2WB	0.8500
C48—H48B	0.9600	O3W—H3WA	0.8501
C48—H48C	0.9600	O3W—H3WC	0.8501
C49—N21	1.309 (6)	O4W—H4WD	0.8500
C49—C50	1.410 (7)	O4W—H4WB	0.8500
C49—H49	0.9300	O5W—H5WA	0.8500
C50—C51	1.366 (7)	O5W—H5WB	0.8501
C50—C61	1.521 (7)	O6W—H6WB	0.8500
C51—C52	1.459 (6)	O6W—H6WC	0.8500
			
N1—C1—W1	178.0 (5)	H66A—C66—H66C	109.5
N2—C2—W1	178.6 (5)	H66B—C66—H66C	109.5
N3—C3—W1	178.5 (5)	O1—C67—N23	125.9 (6)
N4—C4—W1	175.9 (4)	O1—C67—H67	117.0
N5—C5—W1	175.8 (5)	N23—C67—H67	117.0
N6—C6—W1	179.3 (5)	O2—C68—H68A	109.5
N7—C7—W1	177.7 (5)	O2—C68—H68B	109.5
N8—C8—W1	176.7 (5)	H68A—C68—H68B	109.5
N9—C9—W2	179.4 (5)	O2—C68—H68C	109.5
N10—C10—W2	178.0 (5)	H68A—C68—H68C	109.5
N11—C11—W2	176.5 (4)	H68B—C68—H68C	109.5
N12—C12—W2	178.4 (5)	N24—C69—H69A	109.5
N13—C13—W2	175.7 (5)	N24—C69—H69B	109.5
N14—C14—W2	177.3 (5)	H69A—C69—H69B	109.5
N15—C15—W2	177.7 (5)	N24—C69—H69C	109.5
N16—C16—W2	178.0 (4)	H69A—C69—H69C	109.5
N17—C17—C18	123.2 (5)	H69B—C69—H69C	109.5
N17—C17—H17	118.4	N24—C70—H70A	109.5
C18—C17—H17	118.4	N24—C70—H70B	109.5
C19—C18—C17	117.8 (5)	H70A—C70—H70B	109.5
C19—C18—C29	122.9 (5)	N24—C70—H70C	109.5
C17—C18—C29	119.2 (5)	H70A—C70—H70C	109.5
C18—C19—C20	119.6 (4)	H70B—C70—H70C	109.5
C18—C19—C30	120.4 (5)	O3—C71—N24	123.4 (5)
C20—C19—C30	119.9 (5)	O3—C71—H71	118.3
C21—C20—C19	123.6 (5)	N24—C71—H71	118.3
C21—C20—C28	118.7 (5)	O4—C72—H72A	109.5
C19—C20—C28	117.5 (5)	O4—C72—H72B	109.5
C22—C21—C20	121.7 (5)	H72A—C72—H72B	109.5
C22—C21—H21	119.1	O4—C72—H72C	109.5
C20—C21—H21	119.1	H72A—C72—H72C	109.5
C21—C22—C23	123.3 (6)	H72B—C72—H72C	109.5
C21—C22—H22	118.3	N25—C73—H73A	109.5
C23—C22—H22	118.3	N25—C73—H73B	109.5
C24—C23—C22	123.9 (5)	H73A—C73—H73B	109.5
C24—C23—C27	119.1 (4)	N25—C73—H73C	109.5
C22—C23—C27	116.7 (5)	H73A—C73—H73C	109.5
C25—C24—C23	117.9 (5)	H73B—C73—H73C	109.5
C25—C24—C31	120.4 (5)	N25—C74—H74A	109.5
C23—C24—C31	121.7 (5)	N25—C74—H74B	109.5
C24—C25—C26	118.5 (5)	H74A—C74—H74B	109.5
C24—C25—C32	124.1 (5)	N25—C74—H74C	109.5
C26—C25—C32	117.3 (5)	H74A—C74—H74C	109.5
N18—C26—C25	122.4 (5)	H74B—C74—H74C	109.5
N18—C26—H26	118.8	O5—C75—N25	126.7 (5)
C25—C26—H26	118.8	O5—C75—H75	116.7
N18—C27—C28	118.6 (5)	N25—C75—H75	116.7
N18—C27—C23	121.8 (5)	O6—C76—H76A	109.5
C28—C27—C23	119.5 (5)	O6—C76—H76B	109.5
N17—C28—C27	117.8 (4)	H76A—C76—H76B	109.5
N17—C28—C20	122.3 (5)	O6—C76—H76C	109.5
C27—C28—C20	119.9 (5)	H76A—C76—H76C	109.5
C18—C29—H29A	109.5	H76B—C76—H76C	109.5
C18—C29—H29B	109.5	O1—Mn1—N1	90.22 (15)
H29A—C29—H29B	109.5	O1—Mn1—N8^i^	90.16 (15)
C18—C29—H29C	109.5	N1—Mn1—N8^i^	94.90 (14)
H29A—C29—H29C	109.5	O1—Mn1—O2	173.74 (14)
H29B—C29—H29C	109.5	N1—Mn1—O2	87.02 (14)
C19—C30—H30A	109.5	N8^i^—Mn1—O2	84.48 (14)
C19—C30—H30B	109.5	O1—Mn1—N18	96.27 (15)
H30A—C30—H30B	109.5	N1—Mn1—N18	97.25 (15)
C19—C30—H30C	109.5	N8^i^—Mn1—N18	166.21 (14)
H30A—C30—H30C	109.5	O2—Mn1—N18	89.66 (14)
H30B—C30—H30C	109.5	O1—Mn1—N17	93.12 (15)
C24—C31—H31A	109.5	N1—Mn1—N17	170.42 (16)
C24—C31—H31B	109.5	N8^i^—Mn1—N17	94.07 (14)
H31A—C31—H31B	109.5	O2—Mn1—N17	90.49 (14)
C24—C31—H31C	109.5	N18—Mn1—N17	73.47 (15)
H31A—C31—H31C	109.5	O3—Mn2—N9	88.29 (15)
H31B—C31—H31C	109.5	O3—Mn2—O4	174.77 (14)
C25—C32—H32A	109.5	N9—Mn2—O4	86.86 (16)
C25—C32—H32B	109.5	O3—Mn2—N2	86.21 (15)
H32A—C32—H32B	109.5	N9—Mn2—N2	97.96 (16)
C25—C32—H32C	109.5	O4—Mn2—N2	96.40 (15)
H32A—C32—H32C	109.5	O3—Mn2—N19	98.63 (15)
H32B—C32—H32C	109.5	N9—Mn2—N19	167.68 (17)
N19—C33—C34	123.9 (5)	O4—Mn2—N19	85.80 (16)
N19—C33—H33	118.0	N2—Mn2—N19	92.69 (15)
C34—C33—H33	118.0	O3—Mn2—N20	87.44 (15)
C35—C34—C33	118.8 (5)	N9—Mn2—N20	97.24 (15)
C35—C34—C45	121.2 (5)	O4—Mn2—N20	91.22 (14)
C33—C34—C45	119.9 (5)	N2—Mn2—N20	163.32 (15)
C34—C35—C36	119.1 (5)	N19—Mn2—N20	73.05 (14)
C34—C35—C46	120.1 (5)	O5—Mn3—N16^ii^	92.69 (15)
C36—C35—C46	120.7 (5)	O5—Mn3—N10	85.73 (16)
C37—C36—C35	124.2 (5)	N16^ii^—Mn3—N10	98.12 (16)
C37—C36—C44	118.1 (5)	O5—Mn3—O6	169.83 (14)
C35—C36—C44	117.7 (5)	N16^ii^—Mn3—O6	87.63 (15)
C36—C37—C38	125.3 (5)	N10—Mn3—O6	84.16 (16)
C36—C37—H37	117.4	O5—Mn3—N22	99.45 (15)
C38—C37—H37	117.4	N16^ii^—Mn3—N22	92.17 (15)
C37—C38—C39	118.3 (5)	N10—Mn3—N22	168.27 (15)
C37—C38—H38	120.9	O6—Mn3—N22	90.68 (15)
C39—C38—H38	120.9	O5—Mn3—N21	96.53 (15)
C43—C39—C40	119.2 (5)	N16^ii^—Mn3—N21	164.33 (15)
C43—C39—C38	118.4 (4)	N10—Mn3—N21	95.18 (15)
C40—C39—C38	122.4 (5)	O6—Mn3—N21	85.51 (15)
C41—C40—C39	117.7 (5)	N22—Mn3—N21	73.86 (15)
C41—C40—C47	121.9 (5)	C1—N1—Mn1	174.2 (4)
C39—C40—C47	120.3 (5)	C2—N2—Mn2	157.7 (4)
C40—C41—C42	119.8 (5)	C8—N8—Mn1^i^	178.5 (4)
C40—C41—C48	121.8 (5)	C9—N9—Mn2	168.2 (4)
C42—C41—C48	118.4 (5)	C10—N10—Mn3	175.6 (4)
N20—C42—C41	122.1 (5)	C16—N16—Mn3^ii^	171.7 (5)
N20—C42—H42	118.9	C17—N17—C28	119.5 (4)
C41—C42—H42	118.9	C17—N17—Mn1	126.1 (4)
N20—C43—C39	122.1 (4)	C28—N17—Mn1	114.4 (3)
N20—C43—C44	118.1 (4)	C26—N18—C27	120.1 (4)
C39—C43—C44	119.7 (4)	C26—N18—Mn1	124.4 (4)
N19—C44—C36	123.9 (4)	C27—N18—Mn1	115.2 (3)
N19—C44—C43	115.9 (4)	C33—N19—C44	116.4 (4)
C36—C44—C43	120.2 (4)	C33—N19—Mn2	125.7 (4)
C34—C45—H45A	109.5	C44—N19—Mn2	117.4 (3)
C34—C45—H45B	109.5	C42—N20—C43	118.8 (4)
H45A—C45—H45B	109.5	C42—N20—Mn2	126.0 (3)
C34—C45—H45C	109.5	C43—N20—Mn2	115.2 (3)
H45A—C45—H45C	109.5	C49—N21—C60	121.0 (4)
H45B—C45—H45C	109.5	C49—N21—Mn3	125.5 (3)
C35—C46—H46A	109.5	C60—N21—Mn3	113.0 (3)
C35—C46—H46B	109.5	C58—N22—C59	118.2 (4)
H46A—C46—H46B	109.5	C58—N22—Mn3	126.9 (3)
C35—C46—H46C	109.5	C59—N22—Mn3	114.2 (3)
H46A—C46—H46C	109.5	C67—N23—C65	119.4 (5)
H46B—C46—H46C	109.5	C67—N23—C66	123.1 (5)
C40—C47—H47A	109.5	C65—N23—C66	116.8 (5)
C40—C47—H47B	109.5	C71—N24—C70	121.4 (5)
H47A—C47—H47B	109.5	C71—N24—C69	122.3 (5)
C40—C47—H47C	109.5	C70—N24—C69	116.3 (5)
H47A—C47—H47C	109.5	C75—N25—C74	121.0 (5)
H47B—C47—H47C	109.5	C75—N25—C73	124.2 (5)
C41—C48—H48A	109.5	C74—N25—C73	114.2 (5)
C41—C48—H48B	109.5	C67—O1—Mn1	123.5 (4)
H48A—C48—H48B	109.5	C68—O2—Mn1	123.7 (3)
C41—C48—H48C	109.5	C68—O2—H2B	106.5
H48A—C48—H48C	109.5	Mn1—O2—H2B	106.5
H48B—C48—H48C	109.5	C71—O3—Mn2	127.4 (3)
N21—C49—C50	122.2 (5)	C72—O4—Mn2	127.3 (4)
N21—C49—H49	118.9	C72—O4—H4B	105.9
C50—C49—H49	118.9	Mn2—O4—H4B	105.2
C51—C50—C49	119.5 (5)	C75—O5—Mn3	131.7 (3)
C51—C50—C61	122.7 (5)	C76—O6—Mn3	120.4 (3)
C49—C50—C61	117.8 (5)	C76—O6—H6B	106.9
C50—C51—C52	118.7 (4)	Mn3—O6—H6B	107.0
C50—C51—C62	121.3 (5)	H1WA—O1W—H1WC	109.5
C52—C51—C62	120.0 (4)	H2WA—O2W—H2WB	109.5
C53—C52—C60	120.0	H3WA—O3W—H3WC	109.5
C53—C52—C51	121.7 (3)	H4WD—O4W—H4WB	109.5
C60—C52—C51	118.3 (3)	H5WA—O5W—H5WB	109.5
C54—C53—C52	120.0	H6WB—O6W—H6WC	109.5
C54—C53—H53	120.0	C3—W1—C2	74.74 (18)
C52—C53—H53	120.0	C3—W1—C4	101.0 (2)
C53—C54—C55	120.0	C2—W1—C4	72.33 (18)
C53—C54—H54	120.0	C3—W1—C6	144.32 (19)
C55—C54—H54	120.0	C2—W1—C6	74.05 (18)
C59—C55—C54	120.0	C4—W1—C6	85.6 (2)
C59—C55—C56	118.0 (3)	C3—W1—C8	71.71 (19)
C54—C55—C56	122.0 (3)	C2—W1—C8	126.01 (19)
N22—C59—C55	122.6 (3)	C4—W1—C8	74.08 (19)
N22—C59—C60	117.3 (3)	C6—W1—C8	142.57 (18)
C55—C59—C60	120.0	C3—W1—C7	78.1 (2)
N21—C60—C59	119.6 (3)	C2—W1—C7	73.20 (19)
N21—C60—C52	120.4 (3)	C4—W1—C7	144.41 (19)
C59—C60—C52	120.0	C6—W1—C7	76.7 (2)
C57—C56—C55	119.0 (4)	C8—W1—C7	135.9 (2)
C57—C56—C63	122.0 (5)	C3—W1—C1	145.66 (19)
C55—C56—C63	119.0 (4)	C2—W1—C1	135.46 (19)
C56—C57—C58	118.1 (5)	C4—W1—C1	79.52 (19)
C56—C57—C64	123.4 (5)	C6—W1—C1	69.94 (19)
C58—C57—C64	118.4 (5)	C8—W1—C1	75.59 (18)
N22—C58—C57	123.9 (5)	C7—W1—C1	121.09 (19)
N22—C58—H58	118.0	C3—W1—C5	86.2 (2)
C57—C58—H58	118.0	C2—W1—C5	142.7 (2)
C50—C61—H61A	109.5	C4—W1—C5	143.97 (19)
C50—C61—H61B	109.5	C6—W1—C5	109.0 (2)
H61A—C61—H61B	109.5	C8—W1—C5	74.9 (2)
C50—C61—H61C	109.5	C7—W1—C5	71.6 (2)
H61A—C61—H61C	109.5	C1—W1—C5	75.3 (2)
H61B—C61—H61C	109.5	C13—W2—C15	145.16 (19)
C51—C62—H62A	109.5	C13—W2—C11	70.73 (18)
C51—C62—H62B	109.5	C15—W2—C11	143.17 (18)
H62A—C62—H62B	109.5	C13—W2—C14	81.68 (18)
C51—C62—H62C	109.5	C15—W2—C14	108.03 (18)
H62A—C62—H62C	109.5	C11—W2—C14	79.00 (18)
H62B—C62—H62C	109.5	C13—W2—C12	75.69 (19)
C56—C63—H63A	109.5	C15—W2—C12	78.12 (19)
C56—C63—H63B	109.5	C11—W2—C12	117.69 (18)
H63A—C63—H63B	109.5	C14—W2—C12	144.27 (18)
C56—C63—H63C	109.5	C13—W2—C16	139.58 (19)
H63A—C63—H63C	109.5	C15—W2—C16	74.13 (19)
H63B—C63—H63C	109.5	C11—W2—C16	73.61 (19)
C57—C64—H64A	109.5	C14—W2—C16	73.39 (18)
C57—C64—H64B	109.5	C12—W2—C16	139.65 (19)
H64A—C64—H64B	109.5	C13—W2—C9	74.57 (19)
C57—C64—H64C	109.5	C15—W2—C9	76.4 (2)
H64A—C64—H64C	109.5	C11—W2—C9	138.27 (19)
H64B—C64—H64C	109.5	C14—W2—C9	73.65 (19)
N23—C65—H65A	109.5	C12—W2—C9	73.89 (18)
N23—C65—H65B	109.5	C16—W2—C9	125.42 (18)
H65A—C65—H65B	109.5	C13—W2—C10	112.44 (19)
N23—C65—H65C	109.5	C15—W2—C10	79.54 (19)
H65A—C65—H65C	109.5	C11—W2—C10	75.67 (18)
H65B—C65—H65C	109.5	C14—W2—C10	144.3 (2)
N23—C66—H66A	109.5	C12—W2—C10	71.0 (2)
N23—C66—H66B	109.5	C16—W2—C10	75.58 (19)
H66A—C66—H66B	109.5	C9—W2—C10	140.6 (2)
N23—C66—H66C	109.5		
			
N17—C17—C18—C19	−3.2 (8)	C49—C50—C51—C62	−177.7 (5)
N17—C17—C18—C29	175.0 (5)	C61—C50—C51—C62	1.8 (8)
C17—C18—C19—C20	0.5 (7)	C50—C51—C52—C53	−179.3 (4)
C29—C18—C19—C20	−177.7 (5)	C62—C51—C52—C53	−2.6 (6)
C17—C18—C19—C30	−175.5 (5)	C50—C51—C52—C60	1.0 (6)
C29—C18—C19—C30	6.3 (8)	C62—C51—C52—C60	177.7 (4)
C18—C19—C20—C21	179.0 (5)	C60—C52—C53—C54	0.0
C30—C19—C20—C21	−5.0 (8)	C51—C52—C53—C54	−179.7 (4)
C18—C19—C20—C28	2.7 (7)	C52—C53—C54—C55	0.0
C30—C19—C20—C28	178.7 (5)	C53—C54—C55—C59	0.0
C19—C20—C21—C22	−177.6 (5)	C53—C54—C55—C56	−179.7 (4)
C28—C20—C21—C22	−1.3 (8)	C54—C55—C59—N22	−176.7 (4)
C20—C21—C22—C23	4.1 (9)	C56—C55—C59—N22	3.0 (4)
C21—C22—C23—C24	−179.4 (6)	C54—C55—C59—C60	0.0
C21—C22—C23—C27	−5.0 (8)	C56—C55—C59—C60	179.7 (4)
C22—C23—C24—C25	178.4 (5)	N22—C59—C60—N21	−3.1 (4)
C27—C23—C24—C25	4.1 (8)	C55—C59—C60—N21	−180.0 (4)
C22—C23—C24—C31	−2.6 (8)	N22—C59—C60—C52	176.9 (4)
C27—C23—C24—C31	−177.0 (5)	C55—C59—C60—C52	0.0
C23—C24—C25—C26	−0.6 (8)	C53—C52—C60—N21	180.0 (4)
C31—C24—C25—C26	−179.6 (5)	C51—C52—C60—N21	−0.3 (4)
C23—C24—C25—C32	177.2 (5)	C53—C52—C60—C59	0.0
C31—C24—C25—C32	−1.8 (9)	C51—C52—C60—C59	179.7 (4)
C24—C25—C26—N18	−2.0 (8)	C59—C55—C56—C57	−5.4 (6)
C32—C25—C26—N18	−180.0 (5)	C54—C55—C56—C57	174.4 (4)
C24—C23—C27—N18	−5.4 (8)	C59—C55—C56—C63	175.1 (4)
C22—C23—C27—N18	179.9 (5)	C54—C55—C56—C63	−5.1 (6)
C24—C23—C27—C28	177.9 (5)	C55—C56—C57—C58	3.9 (7)
C22—C23—C27—C28	3.1 (7)	C63—C56—C57—C58	−176.6 (5)
N18—C27—C28—N17	2.4 (7)	C55—C56—C57—C64	−178.7 (5)
C23—C27—C28—N17	179.2 (5)	C63—C56—C57—C64	0.8 (8)
N18—C27—C28—C20	−177.5 (5)	C56—C57—C58—N22	0.1 (8)
C23—C27—C28—C20	−0.7 (8)	C64—C57—C58—N22	−177.5 (5)
C21—C20—C28—N17	179.8 (5)	C18—C17—N17—C28	2.4 (8)
C19—C20—C28—N17	−3.7 (8)	C18—C17—N17—Mn1	179.3 (4)
C21—C20—C28—C27	−0.3 (8)	C27—C28—N17—C17	−178.7 (5)
C19—C20—C28—C27	176.2 (5)	C20—C28—N17—C17	1.2 (8)
N19—C33—C34—C35	2.9 (9)	C27—C28—N17—Mn1	4.1 (6)
N19—C33—C34—C45	−179.9 (5)	C20—C28—N17—Mn1	−176.1 (4)
C33—C34—C35—C36	−1.9 (9)	C25—C26—N18—C27	0.9 (8)
C45—C34—C35—C36	−179.0 (5)	C25—C26—N18—Mn1	−171.3 (4)
C33—C34—C35—C46	−178.6 (5)	C28—C27—N18—C26	179.5 (5)
C45—C34—C35—C46	4.2 (9)	C23—C27—N18—C26	2.8 (8)
C34—C35—C36—C37	−177.8 (5)	C28—C27—N18—Mn1	−7.6 (6)
C46—C35—C36—C37	−1.1 (9)	C23—C27—N18—Mn1	175.7 (4)
C34—C35—C36—C44	1.2 (8)	C34—C33—N19—C44	−2.9 (8)
C46—C35—C36—C44	177.9 (5)	C34—C33—N19—Mn2	−175.0 (4)
C35—C36—C37—C38	178.3 (5)	C36—C44—N19—C33	2.1 (8)
C44—C36—C37—C38	−0.7 (8)	C43—C44—N19—C33	−179.3 (5)
C36—C37—C38—C39	1.6 (9)	C36—C44—N19—Mn2	174.8 (4)
C37—C38—C39—C43	−0.9 (8)	C43—C44—N19—Mn2	−6.6 (6)
C37—C38—C39—C40	179.2 (5)	C41—C42—N20—C43	1.7 (8)
C43—C39—C40—C41	−0.1 (8)	C41—C42—N20—Mn2	−177.1 (4)
C38—C39—C40—C41	179.8 (5)	C39—C43—N20—C42	2.3 (7)
C43—C39—C40—C47	−176.9 (5)	C44—C43—N20—C42	179.8 (4)
C38—C39—C40—C47	3.0 (8)	C39—C43—N20—Mn2	−178.8 (4)
C39—C40—C41—C42	3.9 (8)	C44—C43—N20—Mn2	−1.3 (6)
C47—C40—C41—C42	−179.4 (5)	C50—C49—N21—C60	0.3 (8)
C39—C40—C41—C48	−177.6 (5)	C50—C49—N21—Mn3	−170.8 (4)
C47—C40—C41—C48	−0.8 (8)	C59—C60—N21—C49	179.7 (4)
C40—C41—C42—N20	−4.9 (8)	C52—C60—N21—C49	−0.3 (6)
C48—C41—C42—N20	176.5 (5)	C59—C60—N21—Mn3	−8.2 (4)
C40—C39—C43—N20	−3.1 (8)	C52—C60—N21—Mn3	171.80 (18)
C38—C39—C43—N20	176.9 (5)	C57—C58—N22—C59	−2.6 (7)
C40—C39—C43—C44	179.4 (5)	C57—C58—N22—Mn3	167.4 (4)
C38—C39—C43—C44	−0.5 (7)	C55—C59—N22—C58	0.9 (6)
C37—C36—C44—N19	177.8 (5)	C60—C59—N22—C58	−175.9 (4)
C35—C36—C44—N19	−1.3 (8)	C55—C59—N22—Mn3	−170.35 (18)
C37—C36—C44—C43	−0.8 (7)	C60—C59—N22—Mn3	12.8 (4)
C35—C36—C44—C43	−179.8 (5)	O1—C67—N23—C65	−12.7 (10)
N20—C43—C44—N19	5.2 (7)	O1—C67—N23—C66	177.2 (7)
C39—C43—C44—N19	−177.3 (5)	O3—C71—N24—C70	−175.5 (5)
N20—C43—C44—C36	−176.2 (5)	O3—C71—N24—C69	3.2 (8)
C39—C43—C44—C36	1.4 (7)	O5—C75—N25—C74	8.6 (9)
N21—C49—C50—C51	0.4 (8)	O5—C75—N25—C73	179.7 (5)
N21—C49—C50—C61	−179.1 (5)	N23—C67—O1—Mn1	−172.9 (5)
C49—C50—C51—C52	−1.0 (7)	N24—C71—O3—Mn2	−164.4 (4)
C61—C50—C51—C52	178.4 (4)	N25—C75—O5—Mn3	−127.9 (5)

Symmetry codes: (i) −*x*, −*y*+2, −*z*; (ii) −*x*+1, −*y*+2, −*z*+1.
